# A phase 1, open-label, dose-escalation trial of oral TSR-011 in patients with advanced solid tumours and lymphomas

**DOI:** 10.1038/s41416-019-0503-9

**Published:** 2019-06-20

**Authors:** Chia-Chi Lin, Hendrik-Tobias Arkenau, Sharon Lu, Jasgit Sachdev, Javier de Castro Carpeño, Monica Mita, Rafal Dziadziuszko, Wu-Chou Su, Dmitri Bobilev, Lorraine Hughes, Jian Chan, Zhi-Yi Zhang, Glen J. Weiss

**Affiliations:** 1 Department of Oncology, National Taiwan University Hospital, Taipei, Taiwan; 2 Department of Medical Oncology, Sarah Cannon Research Institute and University College London, London, UK; 3 Department of Clinical Science, TESARO: A GSK Company, Waltham, MA USA; 4 Department of Hematology and Oncology, HonorHealth Research Institute, Scottsdale, AZ USA; 5 Department of Oncology, Hospital Universitario La Paz, Madrid, Spain; 6 Department of Hematology and Oncology, Cedars-Sinai Medical Center, Los Angeles, CA USA; 7 Department of Oncology and Radiotherapy, Uniwersyteckie Centrum Kliniczne, Gdansk, Poland; 8 Department of Hematology and Oncology, National Cheng Kung University Hospital, Tainan, Taiwan; 9 Department of Medicine, Beth Israel Deaconess Medical Center, Harvard Medical School, Boston, MA USA

**Keywords:** Targeted therapies, Targeted therapies

## Abstract

**Background:**

Anaplastic lymphoma kinase (*ALK*) gene rearrangements are oncogenic drivers in non-small-cell lung cancer (NSCLC). TSR-011 is a dual ALK and tropomyosin-related kinase (TRK) inhibitor, active against ALK inhibitor resistant tumours in preclinical studies. Here, we report the safety, tolerability and recommended phase 2 dose (RP2D) of TSR-011 in patients with relapsed or refractory *ALK*- and *TRK*-positive advanced cancers.

**Methods:**

In this sequential, open-label, phase 1 trial (NCT02048488), patients received doses of 30 mg, escalated to 480 mg every 24 hours (Q24h), followed by an expansion cohort of patients with *ALK*-positive cancers. The primary objective was to evaluate safety and tolerability. Secondary objectives included pharmacokinetics.

**Results:**

TSR-011 320- and 480-mg Q24h doses exceeded the maximum tolerated dose. At the RP2D of 40 mg every 8 hours (Q8h), the most common grade 3–4 treatment-emergent adverse events occurred in 3.2–6.5% of patients. Of 14 ALK inhibitor-naive patients with *ALK*-positive NSCLC, 6 experienced partial responses and 8 had stable disease.

**Conclusions:**

At the RP2D (40 mg Q8h), TSR-011 demonstrated a favourable safety profile with acceptable QTc changes. Limited clinical activity was observed. Based on the competitive ALK inhibitor landscape and benefit/risk considerations, further TSR-011 development was discontinued.

**Clinical trial registration number:**

NCT02048488.

## Background

Constitutive activation of anaplastic lymphoma kinase (ALK) stemming from *ALK* rearrangement is a driving event in a portion of non-small-cell lung cancers (NSCLCs) and several other cancers. Twenty different *ALK* fusion partner genes have been reported across multiple malignancies.^1^ The uncontrolled activity of ALK fusion proteins results in oncogenic signalling in several downstream pathways that regulate proliferation and malignant transformation.^2,3^ The *EML4*–*ALK* translocation detected in NSCLC is a widely recognised *ALK* fusion gene and is estimated to exist in 5% of all NSCLC cases worldwide.^4^

ALK inhibitors are highly effective in *ALK*-positive NSCLC. For example, crizotinib^5^ had better response rates than chemotherapy, with rates of 74% versus 45% and a median progression-free survival of 10.9 versus 7.0 months, respectively.^6^

Despite the efficacy of crizotinib, acquired resistance in treated patients is a major hurdle, with disease progression occurring within ~1 year.^6–8^ Resistance mutations were identified in 20% of patients with disease progression while participating in a clinical trial.^9^ Resistance mechanisms to crizotinib include *ALK* amplification; secondary mutations in the *ALK* kinase domain (e.g. the gatekeeper mutation); mutations that enhance ALK activation (e.g. the *F1174L* mutation); and activation of bypass signalling pathways (e.g. alterations to EGFR and KIT signalling).^10^ More potent next-generation ALK inhibitors (e.g. ceritinib, alectinib, brigatinib and lorlatinib) were developed to overcome resistance mutations^11^ and are approved for advanced *ALK*-positive NSCLC.

TSR-011 is a potent next-generation ALK inhibitor that also targets tropomyosin-related kinase (TRK) family members (TrkA, TrkB and TrkC).^12^ TRK proteins play a role in the development and maturation of the central and peripheral nervous system, as well as in cell survival. TRK fusion proteins are observed in several cancers, including papillary thyroid carcinoma, colorectal adenocarcinoma, human secretory breast carcinoma and glioblastoma.^13^

TSR-011 has potency at nanomolar concentrations against wild-type *ALK* in vitro and in ALK-dependent cellular models, as well as in ALK-dependent tumour growth inhibitory activity in mouse models.^12,14^ In addition, TSR-011 is ~200-fold more potent than crizotinib against the *L1196M ALK* gatekeeper mutant form and is also about 10-fold more potent than crizotinib against the *R1275Q* mutant,^14^ which is one of the most common activating mutations in neuroblastoma.^15^

This study evaluated the safety, tolerability and pharmacokinetic (PK) profile of TSR-011 in relapsed or refractory locally advanced or metastatic cancer, or cancer for which standard therapy was unavailable.

## Methods

### Study design and treatments

This sequential, open-label, nonrandomised, dose-escalation, phase 1 clinical trial (ClinicalTrials.gov identifier: NCT02048488) evaluated the safety and tolerability of TSR-011 immediate-release (IR) formulation in patients with relapsed or refractory locally advanced or metastatic cancer, or cancer for which standard therapy was unavailable. The study was conducted at 15 sites in Poland, Spain, Taiwan, the United States and the United Kingdom. A formulation sub-study was also undertaken to evaluate a controlled-release (CR) formulation of TSR-011 in a similar patient population.

The primary objective of this trial was to determine the dose-limiting toxicity (DLT), maximum tolerated dose (MTD) and recommended phase 2 dose (RP2D) of TSR-011 based on safety, tolerability, PK and exposure–response analyses.

Endpoints included the incidence of DLTs (defined in the ‘Safety' section) in the first 28-day treatment cycle (cycle 1), the frequency and severity of adverse events (AEs) and serious AEs (SAEs) and changes in laboratory values, vital signs, physical examination findings and Eastern Cooperative Oncology Group (ECOG) performance status (PS). Plasma concentration and PK parameters for TSR-011 were also investigated.

The starting dose was 30 mg, which was determined by review of nonclinical toxicokinetic assessments. A treatment cycle comprised 28 days in the absence of disease progression or unacceptable toxicity. Patients received starting doses ranging from 30 mg once every 24 hours (Q24h) to 480 mg Q24h, and escalation proceeded based on a 3 + 3 design.

The occurrence of DLTs in > 1 of six patients in a specific dosage cohort (or at a rate of ≥ 33% if > 6 patients were in a cohort) indicated that the MTD had been exceeded. The subsequent cohort started at a reduced dosing regimen. Additional dosing schedules were once every 12 hours (Q12h) at 30 and 60 mg and once every 8 hours (Q8h) at 20 and 40 mg.

At the end of each 28-day treatment cycle, individual patients within a cohort were eligible for dose escalation (if other criteria were met and judged by the investigator to be medically indicated). Cohort expansion in the case of > 1 DLT was considered if the DLT could reasonably be considered idiopathic or related to disease progression.

A phase 2a study of the TSR-011 IR formulation was planned to treat *ALK*-positive tumours; however, the study was not initiated when the sponsor terminated further development of TSR-011.

### Patients

Eligible patients were aged ≥ 18 years (except in countries where the age of majority is 16 years; e.g. the United Kingdom) and had metastatic or locally advanced solid tumours that did not respond to standard therapy or progressed, despite standard therapy, and had either refused standard therapy or standard therapy was unavailable. Patients had an estimated life expectancy of ≥ 3 months and adequate organ function. In addition, patients had an ECOG PS ≤ 2. Patients were not required to have measurable disease.

Patients who were pregnant or had leukaemia or uncontrolled congestive heart failure were excluded from the study. A protocol amendment that was implemented before patient enrolment expanded the exclusion criteria to include patients with ongoing cardiac dysrhythmias of National Cancer Institute (NCI) Common Terminology Criteria for Adverse Events (CTCAE) grade ≥ 2, atrial fibrillation of any grade, corrected QT interval (QTc) > 450 ms or risk factors for torsade de pointes and those receiving QTc-prolonging medicines. In addition, patients had to stop taking prescription, over the counter, or herbal medications known to be inhibitors or inducers of cytochrome P450 (CYP) 3A4 or 3A5.

Further, a protocol amendment was made that required patients (excluding those in the formulation sub-study) to demonstrate that they had *ALK*- or *TRK*-positive tumours before enrolment. Mutation status was determined by immunohistochemistry, determination of gene mutations or amplification or analysis of gene rearrangements by fluorescence in situ hybridisation. These assays were performed at local laboratories. A total of 52 patients were enrolled after the protocol amendment implementation (30-mg IR Q12h cohort, *n* = 5; 20-mg IR Q8h cohort, *n* = 16; 40-mg IR Q8h cohort, *n* = 31).

This clinical investigation was conducted in compliance with Good Clinical Practice, the Declaration of Helsinki (version 2008) and other applicable regulatory requirements. The protocol was approved by the Institutional Review Board at each participating site, and all patients provided written informed consent before study participation.

### PK analysis

To evaluate PK and establish the RP2D, blood samples were collected from all patients in the phase 1 dose-escalation trial at daily doses of 30–480 mg.

A liquid chromatography–tandem mass spectrometry (LC–MS/MS) method (API 5500, Applied Biosystems, Waltham, MA, USA) using a Gemini C18 column (Phenomenex, Torrance, CA, USA; 50 × 2.0 mm, 3 µm) for the analysis of TSR-011 in K_2_EDTA human plasma was developed and fully validated by Agilux Laboratories (Worcester, MA, USA). Plasma samples were processed through a Biotage (Uppsala, Sweden) ISOLUTE SLE + 200 μL of a 96-well-supported liquid extraction plate. TSR-011 was determined using electrospray ionisation in positive ion mode, with the multiple reaction monitoring transitions of 578/435 for TSR-011 and 596/453 for TSR-012 as its internal standard. The validated range was 0.87–433.25 nmol/L. The assay interbatch precision, expressed as percent coefficients of variation, ranged from 6.6 to 11.7%, and the interbatch accuracy, expressed as percent bias, ranged from –4.7 to 0.5%.

PK parameters were calculated using noncompartmental analysis (Phoenix WinNonlin version 7.0, Pharsight Corporation, Mountain View, CA, USA).

### Safety

The safety and tolerability of TSR-011 were assessed by evaluating vital signs, physical examination findings, ECOG PS, clinical laboratory testing (serum chemistry, haematology and urinalysis), 12-lead electrocardiograms (ECGs), AEs and visual history. Toxicities were graded and recorded according to NCI CTCAE version 4.03. Patients were assessed for DLTs during the first 28-day cycle. A DLT was defined as any grade ≥ 3 nonhaematologic toxicity, according to NCI CTCAE version 4.03.

### Clinical activity

The overall tumour response in all patients with measurable disease was evaluated, using Response Evaluation Criteria in Solid Tumors, version 1.1,^16^ completed at times of radiologic assessment at weeks 4 and 8 after dosing and every 8 weeks thereafter. For patients without measurable disease at study entry, radiologic assessment was done as per investigator discretion.

### QTc assessments

We assessed potential effects of TSR-011 with the hERG (K_Ir_) in vitro test system. Inhibition of TSR-011 hERG, via displacement of dofetilide binding, exhibited a half-maximal inhibitory concentration (IC_50_) of 2.3 µM. A functional assay evaluating TSR-011 confirmed inhibition of the hERG channel, with an IC_50_ value of 0.172 µM. Evaluation of TSR-011 activity against hNav1.5 expressed in HEK-293 cells resulted in an IC_50_ of 7.6 μM, and a functional patch-clamp test indicated that the IC_50_ for calcium channel inhibition was > 10 μM.

Potential in vivo effects on the respiratory, central nervous and cardiovascular systems were evaluated in oral dose studies in rats and dogs. A telemetered dog cardiovascular study performed with oral doses of 3, 10 and 15 mg/kg showed a dose-dependent prolongation of the PR interval, a small and dose-dependent increase in QRS duration at 10 and 15 mg/kg and prolonged QTc at ≥ 3 mg/kg. Increased heart rate was noted at ≥ 3 mg/kg, decreased pulse pressure at ≥ 10 mg/kg and a mild and transient increase in body temperature at 15 mg/kg. All cardiovascular parameters returned to normal by the subsequent dose, indicating reversibility. TSR-011 did not exhibit any effects in a combined modified Irwin and respiratory safety study in rats at oral doses ≤ 60 mg/kg.

During the phase 1 trial, all patients were monitored by ECGs as part of safety evaluations. The effect of TSR-011 on ECG parameters was evaluated following International Council for Harmonisation of Technical Requirements for Pharmaceuticals for Human Use E14 guidelines. Cardiac function (QT, Bazett-corrected QT formula, Fridericia-corrected QT formula [QTcF], PR, QRS, RR and heart rate) was evaluated by a 12-lead ECG with triplicate readouts at scheduled time points, along with changes from baseline to each scheduled time point.

The TSR-011 CR formulation was evaluated in an open-label sub-study. Each patient received a single 30-mg CR dose of TSR-011, administered orally on day 1. The cohort could be expanded to six patients (3 + 3) if the CR formulation had a desirable PK profile compared with the IR formulation (i.e. attenuation of maximum plasma concentration [C_max_] and maintenance of exposure and lowest plasma trough concentration [C_trough_]). This sub-study was terminated after evaluation of PK from four patients, who subsequently rolled over into the main study for continued treatment.

PK samples, collected before dosing and at 0.5, 1, 2, 4, 8, 12, 18, 24, 32, 44, 48, 72, 96 and 120 h after dosing, were analysed as above, using the LC–MS/MS method. Nonparametric superposition modelling (Phoenix WinNonlin software, Pharsight Corporation) based on the PK of TSR-011 30-mg CR was undertaken to project a daily CR tablet strength matching the current clinical exposure of 40 mg three times daily (TID; Q8h).

### Statistical analysis

Analyses were descriptive in nature. No formal statistical tests were performed. Summary tabulations included the number of observations; mean, standard deviation, median, minimum and maximum for continuous variables; and number and percentage per category for categorical data. Percentages were based on the number of patients with available values, unless specified otherwise.

The analysis sets included a safety population comprising all patients who received ≥ 1 dose of study drug; a PK population comprising all patients who received ≥ 1 dose of study drug and had measurable drug concentrations; and a clinical activity population comprising all patients who received ≥ 1 dose of study drug and had ≥ 1 post-baseline assessment of clinical activity. An additional analysis was performed on patients who met the criteria of achieving ≥ 90% study drug compliance during the first treatment cycle.

## Results

### Study populations

Over a duration of 4 years, 82 patients were screened and 72 were enrolled and treated in the phase 1 main study. Sixteen patients received varying doses of TSR-011, ranging from 30 to 480 mg Q24h. After grade 3 QTc prolongation was observed at doses above 320 mg Q24h, fractional dosing was explored to circumvent such AEs. A total of 56 patients were enrolled into fractional IR dose regimen cohorts (30 mg Q12h, *n* = 6; 60 mg Q12h, *n* = 3; 20 mg Q8h, *n* = 16; 40 mg Q8h, *n* = 31, Fig. S1).

An additional four patients were enrolled in the formulation sub-study; all were subsequently rolled over into the main study and treated with 40-mg IR Q8h after completion of cycle 1.

Overall, 65 patients (90.3%) discontinued the study. The most frequent reason for discontinuation was disease progression (46 patients [63.9%]). The other 19 patients discontinued because of an AE (*n* = 3), death (*n* = 4), physician decision (*n* = 8), or decline in clinical status, loss to follow-up, patient inability to attend the final study visit or withdrawal by the patient (*n* = 1 each).

### Baseline characteristics

Patient demographics and baseline characteristics are summarised in Table 1. The mean age of enrolled patients in the main study was 56.9 years (range, 21–87 years). Most patients were female (56.9%), white (68.1%) and had an ECOG PS of 0 (36.1%) or 1 (59.7%). Of 72 patients enrolled, 56 (77.8%) were ALK inhibitor treatment naive and 41 (56.9%) were *ALK* positive.

**Table 1:** Demographics and baseline characteristics (safety population)

	TSR-011 IR formulation		
	20 mg Q8h (*n* *=* 16)	40 mg Q8h (*n* *=* 31)	All patients (*N* *=* 72)
Age, years			
Mean (SD)	54.9 (9.64)	51.7 (13.78)	56.9 (13.44)
Median	53.5	51	58
Min–max	36–68	21–77	21–87
Sex, *n* (%)			
Male	8 (50.0)	16 (51.6)	31 (43.1)
Female	8 (50.0)	15 (48.4)	41 (56.9)
Race, *n* (%)			
Asian	5 (31.3)	13 (41.9)	20 (27.8)
Black or African American	0	1 (3.2)	1 (1.4)
White	10 (62.5)	17 (54.8)	49 (68.1)
Unknown	1 (6.3)	0	2 (2.8)
Smoking history, *n* (%)			
Former	8 (50.0)	15 (48.4)	32 (44.4)
Never	8 (50.0)	16 (51.6)	40 (55.6)
ECOG performance status, *n* (%)			
0	5 (31.3)	13 (41.9)	26 (36.1)
1	10 (62.5)	17 (54.8)	43 (59.7)
2	0	1 (3.2)	2 (2.8)
3	1 (6.3)	0	1 (1.4)
Prior ALK inhibitor treatment, *n* (%)			
Yes^a^	7 (43.8)	8 (25.8)	16 (22.2)
Crizotinib	6 (37.5)	6 (19.4)	12 (16.7)
Ceritinib	0	3 (9.7)	3 (4.2)
Alectinib	1 (6.3)	1 (3.2)	2 (2.8)
No	9 (56.3)	23 (74.2)	56 (77.8)
ALK-positive mutation status,^b^ *n* (%)			
Yes	10^c^ (62.5)	24^d^ (77.4)	41 (56.9)
No	2 (12.5)	4 (12.9)	11 (15.3)
Not done	4 (25.0)	3 (9.7)	20 (27.8)
TRK-positive mutation status, *n* (%)			
Yes	6 (37.5)	3 (9.7)	11 (15.3)
No	6 (37.5)	28 (90.3)	48 (66.7)
Not done	4 (25.0)	0	13 (18.1)

*ALK* anaplastic lymphoma kinase, *ECOG* Eastern Cooperative Oncology Group, *IR* immediate release, *max* maximum, *min* minimum, *Q8h* once every 8 hours, *SD* standard deviation, *TRK* tropomyosin-related kinase

^a^Three patients received two ALK inhibitors

^b^Included gene mutations, amplifications or rearrangements

^c^Three patients were ALK inhibitor naive

^d^Seventeen patients were ALK inhibitor naive

### Safety and tolerability

Overall, patients remained on treatment for a median of 112 days (range, 4–848 days), and 48.6% of patients remained in the study for 2–5 cycles, with 28 days per cycle.

A total of 70 patients (97.2%) in the safety population experienced ≥ 1 treatment-emergent AE (TEAE), as summarised in Table 2. The most frequently reported TEAEs by class, regardless of causality, were gastrointestinal disorders (44 patients [61.1%]), followed by general disorders and administration-site conditions (fatigue, asthenia, disease progression and peripheral oedema; 36 patients [50.0%]). Individual TEAEs reported in > 15% of patients included constipation and fatigue (14 patients [19.4%] each); diarrhoea, vomiting and QTc prolongation (13 patients [18.1%] each); and asthenia, headache, decreased appetite and anaemia (11 patients [15.3%] each).

**Table 2:** Overall TEAEs in all patients of the phase 1 main study and two fractional dose treatment groups

	TSR-011 IR formulation		
	20 mg Q8h (*n* *=* 16)	40 mg Q8h (*n* *=* 31)	All patients (*N* *=* 72)
Total number of TEAEs^a^	159	167	621
Number (%) of patients with			
Any TEAE	16 (100.0)	29 (93.5)	70 (97.2)
Any serious TEAE	7 (43.8)	12 (38.7)	28 (38.9)
Any study drug-related TEAE	9 (56.3)	13 (41.9)	40 (55.6)
Any TEAE leading to treatment discontinuation	2 (12.5)	5 (16.1)	11 (15.3)
Any AE leading to death^b^	2 (12.5)	2 (6.5)	6 (8.3)

*AE* adverse event, *IR* immediate release, *Q8h* once every 8 hours, *TEAE* treatment-emergent adverse event

Note: The total number of AEs counted all TEAEs for patients

^a^At each level of patient summarisation, a patient was counted once if the patient reported one or more event

^b^No AEs leading to death were deemed related to treatment

Most TEAEs (64% of events) were grade 1 in severity. However, grade 3 TEAEs were reported in 37 patients (51.4%; specific grade 3 events reported in > 1 patient are shown in Table 3). One patient experienced a grade 4 TEAE of anaemia.

**Table 3:** Grade 3 treatment-emergent adverse events reported by more than one patient in the Q8h cohorts compared with the safety population

Preferred term	TSR-011 IR formulation, *n* (%)		
	20 mg Q8h (*n* *=* 16)	40 mg Q8h (*n* *=* 31)	All patients (*N* *=* 72)
Anaemia	2 (12.5)	2 (6.5)	5 (6.9)
Electrocardiogram QTc prolonged	0	1 (3.2)	4 (5.6)
Asthenia	1 (6.3)	1 (3.2)	3 (4.2)
Dyspnoea	0	0	3 (4.2)
Ascites	1 (6.3)	0	2 (2.8)
Fatigue	1 (6.3)	0	2 (2.8)
Metastases to the central nervous system	1 (6.3)	1 (3.2)	2 (2.8)

*IR* immediate release, *Q8h* once every 8 hours, *QTc* corrected QT

Note: Adverse events were coded using the *Medical Dictionary for Regulatory Activities*, version 18.0. If the severity of an adverse event was missing, the adverse event was reported as “severe”

Study drug-related TEAEs occurred in 40 patients (55.6%). The most commonly reported study drug-related TEAEs were QTc prolongation, constipation, decreased appetite, vomiting and fatigue (Table 4). One patient (1.4%) experienced a study drug-related SAE.

**Table 4:** Drug-related treatment-emergent adverse events reported by more than one patient in the Q8h cohorts compared with the safety population

Preferred term	TSR-011 IR formulation, *n* (%)		
	20 mg Q8h (*n* *=* 16)	40 mg Q8h (*n* *=* 31)	All patients (*N* = 72)
Electrocardiogram QTc prolonged	1 (6.3)	3 (9.7)	12 (16.7)
Constipation	2 (12.5)	2 (6.5)	5 (6.9)
Decreased appetite	1 (6.3)	0	4 (5.6)
Vomiting	2 (12.5)	1 (3.2)	4 (5.6)
Fatigue	1 (6.3)	0	4 (5.6)
Diarrhoea	1 (6.3)	0	3 (4.2)
Dysgeusia	0	0	3 (4.2)
Nausea	0	1 (3.2)	3 (4.2)
Peripheral oedema	0	1 (3.2)	3 (4.2)
Headache	0	0	3 (4.2)
Abnormal dreams	1 (6.3)	0	2 (2.8)
Anaemia	0	1 (3.2)	2 (2.8)
Asthenia	1 (6.3)	0	2 (2.8)
Dysaesthesia	1 (6.3)	0	2 (2.8)
Hot flush	1 (6.3)	0	2 (2.8)
Insomnia	0	2 (6.5)	2 (2.8)
Myalgia	0	0	2 (2.8)
Rash	0	1 (3.2)	2 (2.8)
Increased transaminases	0	0	2 (2.8)

*IR* immediate release, *Q8h* once every 8 hours, *QTc* corrected QT interval

Note: At each level of patient summarisation, a patient was counted once if the patient reported one or more event. Adverse events were coded using the *Medical Dictionary for Regulatory Activities*, version 18.0

Treatment-emergent SAEs occurred in 28 patients (38.9%) (Table 5). The most frequent SAE among patients with ≥ 1 treatment-emergent SAE was disease progression, which occurred in six patients (8.3%). All treatment-emergent SAEs were considered by the investigator as unrelated or unlikely to be related to the study drug, except for anaemia in one patient that was considered related to the study drug.

**Table 5:** Treatment-emergent SAEs reported by more than one patient by system organ class and the preferred term (safety population)

Preferred term	TSR-011 IR formulation		
	20 mg Q8h (*n* = 16)	40 mg Q8h (*n* = 31)	All patients (*N* = 72)
Total number of treatment-emergent SAEs^a^	14	16	49
Patients with ≥ 1 treatment-emergent SAE, *n* (%)^b^	7 (43.8)	12 (38.7)	28 (38.9)
Nervous system disorders, *n* (%)	1 (6.3)	2 (6.5)	8 (11.1)
Mental impairment, *n* (%)	1 (6.3)	0	2 (2.8)
Seizure	1 (6.3)	1 (3.2)	2 (2.8)
Subarachnoid haemorrhage	0	1 (3.2)	1 (1.4)
General disorders and administration-site conditions, *n* (%)	2 (12.5)	2 (6.5)	7 (9.7)
Disease progression	2 (12.5)	2 (6.5)	6 (8.3)
Asthenia	1 (6.3)	0	1 (1.4)
Gastrointestinal disorders, *n* (%)	1 (6.3)	1 (3.2)	5 (6.9)
Abdominal pain	0	1 (3.2)	1 (1.4)
Gastric haemorrhage	1 (6.3)	0	1 (1.4)
Infections and infestations, *n* (%)	1 (6.3)	4 (12.9)	5 (6.9)
Gastric ulcer helicobacter	0	1 (3.2)	1 (1.4)
Infection	0	1 (3.2)	1 (1.4)
Lower respiratory tract infection	0	1 (3.2)	1 (1.4)
Mastoiditis	1 (6.3)	0	1 (1.4)
Pneumonia	0	1 (3.2)	1 (1.4)
Respiratory, thoracic and mediastinal disorders, *n* (%)	0	1 (3.2)	4 (5.6)
Dyspnoea	0	0	3 (4.2)
Pleural effusion	0	1 (3.2)	1 (1.4)
Neoplasms benign, malignant and unspecified (including cysts and polyps), *n* (%)	2 (12.5)	1 (3.2)	3 (4.2)
Metastases to the central nervous system, *n* (%)	1 (6.3)	1 (3.2)	2 (2.8)
Basal cell carcinoma	1 (6.3)	0	1 (1.4)
Blood and lymphatic system disorders, *n* (%)	0	2 (6.5)	2 (2.8)
Anaemia	0	2 (6.5)	2 (2.8)
Cardiac disorders	0	1 (3.2)	1 (1.4)
Pericardial effusion	0	1 (3.2)	1 (1.4)
Hepatobiliary disorders, *n* (%)	1 (6.3)	0	1 (1.4)
Portal vein thrombosis	1 (6.3)	0	1 (1.4)
Metabolism and nutrition disorders, *n* (%)	1 (6.3)	0	1 (1.4)
Dehydration	1 (6.3)	0	1 (1.4)
Musculoskeletal and connective tissue disorders, *n* (%)	1 (6.3)	0	1 (1.4)
Bone pain	1 (6.3)	0	1 (1.4)
Vascular disorders, *n* (%)	1 (6.3)	0	1 (1.4)
Deep-vein thrombosis	1 (6.3)	0	1 (1.4)

*AE* adverse event, *IR* immediate release, *Q8h* once every 8 hours, *SAE* serious adverse event

Note: AEs were coded using the *Medical Dictionary for Regulatory Activities*, version 18.0

^a^The total number of treatment-emergent SAEs counts all treatment-emergent SAEs for patients

^b^At each level of patient summarisation, a patient was counted once if the patient reported one or more event

Overall, 12 TEAEs led to study drug discontinuation in 11 patients (15.3%). Of these, ECG QTc prolongation (two patients) and pneumonitis (one patient) were considered related to the study drug. Six patients died because of TEAEs, including disease progression (*n* = 5) and subarachnoid haemorrhage, not related to the study drug (*n* = 1). In addition, one patient died due to cardiopulmonary failure before receiving the study drug.

During dose escalation, none of the patients experienced DLTs in the 30-, 60-, 120- and 240-mg IR Q24h cohorts. Grade 3 QTc prolongation occurred in two of four patients in the 480-mg Q24h cohort. Hence, the dose was de-escalated to 320 mg Q24h. One patient in this cohort experienced grade 3 ECG QTc prolongation within the DLT period, and an additional patient in this cohort was retrospectively determined to have experienced grade 3 QTc prolongation.

It was hypothesised that QTc prolongation is related to C_max_, so fractionated dosing was implemented to circumvent QTc prolongation while maintaining the drug trough level. Evaluated cohorts included 30 mg Q12h, 20 mg Q8h, 60 mg Q12h and 40 mg Q8h. Based on the evaluation of QTc prolongations and PK profiles (discussed in the following sections), 40-mg IR TID (Q8h) was determined to be the RP2D.

### PK results

**Fig. 1:**
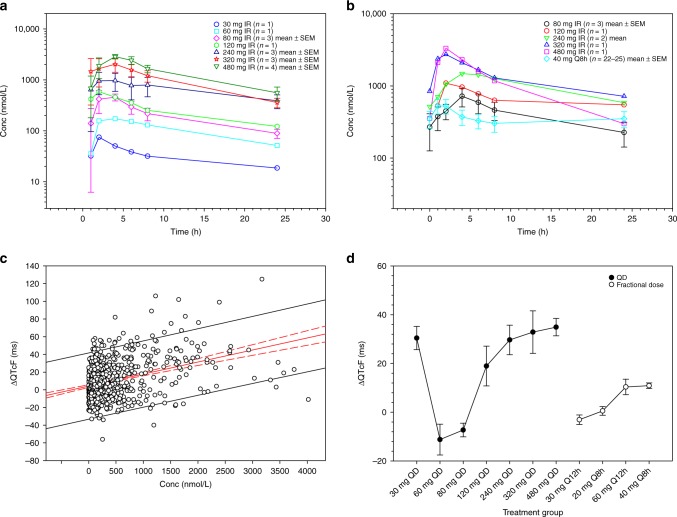
Mean ± SEM plasma concentration–time profiles (semi-log) of TSR-011 following single oral daily doses in patients with cancer. **a** Cycle 1/day 1. **b** Cycle 2/day 1. Summary of ΔQTcF by plasma concentration and dose. **c** PK concentration–response (PK-QT) relationship from all dose groups (30 mg QD, 60 mg QD, 80 mg QD, 120 mg QD, 240 mg QD, 320 mg QD, 480 mg QD, 30 mg Q12h, 20 mg Q8h, 60 mg Q12h, 40 mg Q8h and 30 mg QD controlled release). **d** Plot of dose and regimen versus mean ΔQTcF (ms) from baseline with 90% CI. *ΔQTcF* change in Fridericia-corrected QT interval, *CI* confidence interval, *Conc* concentration, *IR* immediate release, *PK* pharmacokinetics, *Q8h* once every 8 hours, *Q12h* once every 12 hours, *QD* once daily, *SEM* standard error of the mean

Plasma concentration–time profiles (Fig. 1) for both cycle 1/day 1 and cycle 2, day 1/day 29 of TSR-011 were characterised by rapid absorption, with median time to C_max_ (t_max_) occurring 2–4 h after a single oral dose at cycle 1. Following attainment of C_max_, TSR-011 concentrations declined biexponentially. Plasma concentration profiles were comparable across all cohorts, as well as with repeat dosing for 29 days. The half-life ranged from 7 to 27 h, with apparent clearance and apparent volume of distribution of 9.62–46.60 L/h and 188–1620 L, respectively. Systemic exposure increased supraproportionally, with a 41- and 42-fold increase in geometric mean C_max_ and area under the concentration–time curve (AUC) from 0 to 24 h, respectively, over the 16-fold increase in TSR-011 dose (range, 30–480 mg).

Following daily oral administration of TSR-011 (80–480 mg) at cycle 2/day 1, the median t_max_ was achieved between 2.00 and 6.00 h, with C_max_ ranging from 447.11 to 3327.36 nmol/L. Apparent clearance and apparent volume of distribution ranged from 9.26 to 29.10 L/h and 206 to 408 L, respectively.

Systemic exposure at steady state increased sub-proportionally with increasing doses, with a 3.3- and 2.9-fold increase in exposure for C_max_ and AUC from time 0 to the last measurable concentration, respectively, over the sixfold increase in dose (range, 80–480 mg). The accumulation ratio, where calculable, ranged from 0.68 to 5.24.

### QTc prolongation, mitigation strategy and RP2D

QTc prolongation was observed during dose escalation and was the key determinant of the MTD. The relationship between TSR-011 plasma concentration and change from baseline in QTcF (ΔQTcF) was explored using linear regression with prediction intervals (Fig. 1c, solid black lines). All the ΔQTcF data with matching drug concentration values were used for the analysis. The correlation was positive and significant (i.e. slope of 0.02358 [red solid line]; 95% confidence interval [CI], 0.01970–0.02747 [red dotted line]; *P* < 0.0001), indicating that QTc prolongation was drug related.

The mean ΔQTcF appeared to be dose dependent (Fig. 1d). All the available ΔQTcF data from each patient were included in the analysis. Once-daily (QD) doses at 240 mg (*n* = 3), 320 mg (*n* = 3) and 480 mg (*n* = 4) corresponded to a ΔQTcF of > 20 ms (a substantial risk for proarrhythmic disorders, including torsade de pointes). The mean ΔQTcF for 120 mg QD (*n* = 1) was 19 ms, with the upper bound of the two-sided 90% CI at 27 ms. The data from one patient in the 30-mg QD cohort were considered to be outlying. This patient was taking concomitant ondansetron, which has been associated with QTc prolongation. Therefore, the observed QTc prolongation may not be related to TSR-011 at this dose level.

Fractionated dosing was implemented to reduce potential QTc prolongation. Both twice-daily (BID; Q12h) and TID (Q8h) dosing regimens were explored at cumulative daily doses of 60 and 120 mg (Fig. 1d). This strategy maintained mean ΔQTcF below 20 ms for all four dose regimens. The highest mean ΔQTcF of 11 ms (90% CI, 9.75–12.00 ms) was observed in the 40-mg TID cohort (*n* = 27). While no specific guidance exists, it is generally considered acceptable to conclude that a change in QTc from baseline is not clinically meaningful, if the upper bound of the two-sided 90% CI for the ΔQTcF is < 20 ms. At 40-mg TID dosing, there was one observed grade 3 TEAE of QTc prolongation. The dose of 40 mg TID (Q8h) was determined to be the RP2D; dose escalation was stopped.

### CR formulation

#### Rationale for a switch from IR to CR

Fractionated dosing (Q8h) was successful in minimising the ratio of C_max_ to C_trough_ while maintaining the required drug trough level. At the 120-mg QD dose (day 29 of steady state, *n* = 1), this ratio was ~2.00. It was reduced to ~1.42 (day 29 of steady state, *n* *=* 25) with the 40-mg TID (Q8h) dose, which was equivalent to a 120-mg cumulative daily dose. At this dosage and regimen, the overall steady-state PK profile (day 29, *n* = 22–25) exhibited high interpatient variability, with percent coefficients of variation ranging from 108 to 132% and a mean C_max_ and C_trough_ of 528.41  and 351.38 nmol/L, respectively. The PK profiles were also evaluated using the fluctuation index, defined as (C_max_–C_trough_)/C_avg_, and swing percentage, defined as (C_max_–C_trough_)/C_trough_ × 100, with ranges of 0.25–1.60 and 28.8–295.0%, respectively. The CR formulation was under development to mimic the PK profile of 40 mg TID, with the potential to generate a more continuous effect through compliance with the daily dosage routine and maintenance of therapeutic plasma concentrations. Less fluctuation and swing are desirable in a CR formulation through reduced intrapatient variability. The criteria set for the new formulation were such that the mean C_max_/C_trough_ ratio should be maintained within 2.00, with a C_trough_ of about 351.38 nmol/L. No observed concentration should be higher than 2599.50 nmol/L, which was the highest concentration observed in the 40-mg IR TID cohort.

#### PK of CR formulation

A CR formulation was subsequently developed to mimic the PK profile of the 40-mg TID (Q8h) dose, including the mean C_trough_. PK parameters were measured in a cohort of four patients who received a 30-mg CR formulation. The mean C_max_ was 37.95 nmol/L, and t_max_ occurred ~4 h after dosing. Based on nonparametric superposition modelling, consistent with fractionated dosing, the ratio of mean C_max_ to mean C_trough_ was maintained at about 1.65 of steady state with daily dosing. In addition, based on the 30-mg CR prototype, a 240-mg CR daily dose was projected to match the clinical exposure of 40 mg TID (Q8h). The trough level for the 240-mg CR prototype was about 415.92 nmol/L, which matches the trough of 40 mg TID (Q8h) at a mean of 351.38 nmol/L. The mean C_max_ was projected to be < 693.20 nmol/L. Based on the linear regression (Fig. 1c), at 693.20 nmol/L, the mean ΔQTcF with 95% CI ranged from 10.6 to 16.7 ms, which is within the manageable risk of QTc prolongation.

### Efficacy

While tumour response data were collected from 66 patients, here, we present response data only for patients with *ALK*-positive tumour samples (*n* *=* 22) receiving the RP2D (40 mg Q8h). Fourteen of these patients were ALK inhibitor naive, and eight had been previously treated with an ALK inhibitor. No *ALK*-positive patient had a complete response; seven patients had a partial response (six confirmed, one unconfirmed) and 14 had stable disease. Among ALK inhibitor-naive patients, six had partial responses (five confirmed, one unconfirmed) and eight had stable disease (Table S1). The median duration of treatment was 112 days (range, 4–848 days), and 47% of patients remained in the study for 2–5 cycles. Three *ALK*-positive patients are continuing in the study and receiving the study drug as of the time of the study report. A patient with a neuroblastoma harbouring an *ALK* mutation (*R1275Q*) did not respond to TSR-011 treatment.

## Discussion

The PK and safety of TSR-011 were studied in this sequential, open-label, nonrandomised, dose-escalation, phase 1 clinical trial at doses ranging from 20 to 480 mg administered to patients with relapsed or refractory locally advanced or metastatic cancer. The 320- and 480-mg Q24h doses exceeded the MTD. Patients in these cohorts experienced grade 3 ECG QTc prolongation. Fractional dosing in subsequent cohorts resulted in an RP2D of 40 mg TID (Q8h).

Safety data show that the RP2D was generally well tolerated, with an acceptable and manageable AE profile. Compared with AEs of grade ≥ 3 severity reported with the use of alectinib or brigatinib,^17,18^ no grade ≥ 3 pneumonitis or hypoxia was observed in patients treated with TSR-011 (40 mg TID). Longer-term treatment of patients at this dosage suggested that most could continue treatment with dose interruptions or dose reductions to manage TEAEs.

At the 40-mg TID (Q8h) RP2D (*n* *=* 27, QTc-evaluable patients), the frequency of drug-related QTc prolongation was lower compared with the overall safety population (9.7% vs 16.7%, respectively). One in 27 patients (3.7%) had a ΔQTc of > 60 ms and a QTc > 500 ms (a grade 3 event). Clinical trials of ceritinib showed that, among 919 patients, 6% had a ΔQTc of > 60 ms and 1.3% had a QTc > 500 ms.^19^ In clinical trials of alectinib and brigatinib, QTc was not prolonged to any clinically relevant extent.^17,18^

PK analyses showed that after single-dose administration of TSR-011, systemic exposure C_max_ and AUC values increased supraproportionally over the 30- to 480-mg dose range. Profiling of the CYPs responsible for metabolism of TSR-011 was conducted, using a panel of recombinant CYP enzymes and liver microsomes. The results indicated that CYP3A4 is responsible for the majority of CYP-dependent metabolism of TSR-011, with minor amounts of metabolism by other enzymes, including CYP2D6, CYP2C9, and CYP1A. Meanwhile, TSR-011 inhibited CYP3A4 and CYP2D6 in human liver microsomes, with an IC_50_ of 23.3 and 32.9 µM, respectively, which may provide a rationale for the supraproportional systemic exposure observed after administration of a single dose. After repeated dosing, systemic exposure at steady state increased in a sub-proportional manner over the dose range of 80–480 mg. TSR-011 is a substrate of efflux transporters with a P_app_(B–A)/(A–B) ratio of 7.7. It is speculated that with repeated dosing, TSR-011 may induce the transporters as well as CYP enzymes at higher doses. Overall, high interpatient variability in the C_max_ and AUC PK parameters was observed in these cohorts.

To date, five ALK inhibitors (crizotinib, ceritinib, alectinib, brigatinib and lorlatinib) have been approved by the US Food and Drug Administration for treatment of advanced NSCLC harbouring *ALK* aberrations.^5^ Approved ALK inhibitor formulations have less frequent dosing schedules than TSR-011 at Q8h. For example, alectinib and crizotinib are dosed BID and ceritinib is dosed QD.^19^ Therefore, the TSR-011 CR formulation was an attractive line of inquiry. In the formulation sub-study, after a single 30-mg CR dose of TSR-011, C_max_ and AUC values were lower than those observed with a single 30-mg IR dose of TSR-011. There was high interpatient variability in the C_max_ and AUC parameters. Nonparametric superposition projected that 240 mg of this CR would have C_trough_ of about 415.92 nmol/L, which matches the 40 mg IR TID cohort, with a C_max_ of about 693.20 nmol/L, at which QTc prolongation is manageable.

At the RP2D, TSR-011 demonstrated clinical activity in the *ALK*-positive NSCLC subgroup that included both ALK inhibitor-naive patients and those previously treated with an ALK inhibitor. A higher response rate was observed in ALK inhibitor-naive patients (partial response rate, 42.9% vs 12.5%, respectively). Stable disease occurred in eight ALK inhibitor-naive patients (57.1%) and six patients previously treated with an ALK inhibitor (75.0%). The observed efficacy of TSR-011 at the RP2D appears to be significantly less than that of other ALK inhibitors, which have shown an objective response rate of > 70% in a similar population. Similarly, an objective response rate of 12.5% is notably lower in the ALK pre-treated setting than what has been reported with all next-generation ALK inhibitors post crizotinib (55%, ceritinib; 38–48%, alectinib; 45–54%, brigatinib; 48%, lorlatinib). This was due to a suboptimal RP2D, which was limited by QTc prolongation. In preclinical studies, mice treated with an oral dose of TSR-011 60 mg/kg showed complete ALK inhibition in Karpas-299 tumours at 8 h, with a plasma concentration of 698.00 nmol/L. Oral daily dosing of 60 mg/kg resulted in > 80% tumour growth inhibition, with an overall 24-h exposure of 15,500 ng×h/mL. A similar drug exposure for humans would require a daily dose ranging from 150 to 720 mg, based on the apparent clearance of 9.62–46.60 L/h. Exposure of the RP2D at 40 mg TID (Q8h) (Fig. 1b) was lower than that of 120 mg IR QD. The high interpatient variability of PK was not favourable for the further development of the compound.

In conclusion, dose escalation of TSR-011 in patients with relapsed or refractory cancers established the RP2D of 40 mg TID (Q8h). QTc prolongation was a DLT with Q24h dosing at or above 320 mg. At the RP2D, TSR-011 had a favourable safety profile compared with most approved ALK inhibitors. Based on the competitive ALK inhibitor landscape and DLT of QTc prolongation, the development of TSR-011 has been discontinued.

## Supplementary information

Table S1

## Data Availability

The data on internal records will be provided upon request.
