# Supplementary material for: A phase 1, open-label, dose-escalation trial of oral TSR-011 in patients with advanced solid tumours and lymphomas
Source: Br J Cancer. 2019 Jun 20;121(2):131–8. doi: 10.1038/s41416-019-0503-9 (PMC6738096; doi:10.1038/s41416-019-0503-9)
Supplement: Supplementary file 1 — Table S1 [file 41416_2019_503_MOESM1_ESM.docx]

**SUPPLEMENTARY INFORMATION**

**Table S1.** Best overall response^a^ in all patients with *ALK*-positive non-small-cell lung cancer who received TSR-011 40 mg every 8 hours

|  | ALK inhibitor-naïve  (*n* = 14) | Prior ALK inhibitor-treated (*n* = 8) | All patients (*N* = 22) |
| --- | --- | --- | --- |
| Best overall response, *n* (%) |  |  |  |
| Complete response | 0 | 0 | 0 |
| Partial response | 6 (42.9) | 1 (12.5) | 7 (31.8) |
| Stable disease | 8 (57.1) | 6 (75.0) | 14 (63.6) |
| Progressive disease | 0 | 1 (12.5) | 1 (4.5) |
| Objective response, *n* (%) |  |  |  |
| Complete response + partial response | 6 (42.9) | 1 (12.5) | 7 (31.8) |

*ALK*=anaplastic lymphoma kinase.

^a^Includes confirmed and unconfirmed responses

**Fig S1.** Enrollment and Outcomes





*ALK*=anaplastic lymphoma kinase, *CR*=controlled-release, *IR*=immediate release, *PK*=pharmacokinetic, *Q8h*=once every 8 hours, *Q12h*=once every 12 hours, *Q24h*=once every 24 hours.

^a^Safety population included all patients who received at least 1 dose of study drug.

^b^PK population included all patients who received at least 1 dose of study drug and had measureable drug concentration. The PK population could exclude patients if their derived PK parameters were considered invalid due to relevant missing values.

^c^Clinical activity population consisted of all patients who received at least 1 dose of study drug and had at least 1 post baseline assessment of clinical activity.

**Table S2.** List of Institutions

| Site # | Investigator | Address |
| --- | --- | --- |
| 1102 | Jasgit Sachdev, MD | Scottsdale Healthcare Hospitals  10510 N. 92nd Street, Ste. 200  Scottsdale, AZ 85258  United States |
| 1103 | Stephen P. Anthony, DO | Evergreen Hematology and Oncology  309 E. Farwell Road, Ste. 100  Spokane, WA 99218  United States |
| 1104 | Monica Mita, MD | Cedars Sinai Medical Center  8700 Beverly Blvd.  Los Angeles, CA 90048  United States |
| 1109 | Jeffrey Infante, MD | The Sarah Cannon Research Institute  250 25th Ave. North, Ste 200  Nashville, TN 37203  United States |
| 1110 | Glen J. Weiss, MD, MBA | Western Regional Medical Center, Inc  14200 W. Celebrate Life Way  Goodyear, AZ 85338  United States |
| 1111 | Paul R. Conkling, MD | Virginia Oncology Associates  5900 Lake Wright Drive  Norfolk, VA 23502  United States |
| 34200 | Javier de Castro Carpeño | Hospital Universitario La Paz,  Servecio de Oncologia Medica  Paseo de la Catellana, 261  Madrid 28046  Spain |
| 34201 | Rafael Lopez | Hospital Clinico Universitario de  Santiago de Compostela, Servicio de  Oncologia, C/A Choupana s/n  Santiago de Compostela, A Coruna 15706  Spain |
| 44111 | Hendrik-Tobias Arkenau, Dr | Sarah Cannon Research Institute UK  93 Harley Street  London W1G 6AD  United Kingdom |
| 48101 | Rodryg Ramlau, MD, PhD | Med-Polonia Sp. z o.o.  Obornicka 262  Poznan, Weilkopolskie, 60-693  Poland |

| 48102 | Andrzej Kazarnowicz, MD | Samodzielny Publiczny Zespol  Gruzlicy i Chorob Pluc, Oddzial  Onkologii z Pododzialem  Chemioterapii Nowotworow Pluc  Jagiellonska 78  Olsztyn, Warminsko-mazurskie 10-357  Poland |
| --- | --- | --- |
| 48103 | Katarzyna Zajda, MD | Centrum Onkologii Instytut im. Marii  Sklodowskiej-Curie, Klinika  Nowotworow Pluca I Klatki  Piersiowej  Roentgena 5  Warsaw, Mazowieckie 02-781  Poland |
| 48104 | Rafal Dziadziuszko, MD, PhD | Uniwersyteckie Centrum Kliniczne,  Klinika Onkologii i Radioterapii  Debinki 7  Gdansk, Pomorskie 80-952  Poland |
| 886001 | Chia-Chi Lin | National Taiwan University Hospital  7 Chung-Shan South Road  Taipei, Taiwan 10002 |
| 886002 | Wu-Chou Su, MD | National Cheng Kung University  Hospital  138 Sheng-Li Rd  Tainan, Taiwan, R.O.C. 704 |
